# A Review of the Uses and Medicinal Properties of *Dennettia tripetala* (Pepperfruit)

**DOI:** 10.3390/medsci3040104

**Published:** 2015-11-02

**Authors:** Sylvia Oghogho Iseghohi

**Affiliations:** Department of Biochemistry, University of Benin, P.M.B. Benin 1154, Nigeria; E-Mail: sylvia.iseghohi.13@ucl.ac.uk

**Keywords:** *Dennettia tripetala*, Pepperfruit, antioxidant, antimicrobial, analgesic, food preservation

## Abstract

*Dennettia tripetala* (commonly known as Pepperfruit) is widely consumed by the inhabitants of West Africa due to its distinctive spicy taste. It is also used traditionally as a remedy for cough, fever, toothache, diabetes, and nausea. The highly nutritious fruit is rich in protein, carbohydrates, as well as the antioxidant vitamins A, C, and E. The plant possesses phytochemicals that have been shown to elicit antimicrobial, insecticidal, analgesic, and anti-inflammatory properties. The plant has also been shown to possess chemotherapeutic, antihyperglycemic, and antioxidant properties. In addition, *D. tripetala* finds application in food preservation and seasoning. This review is the first attempt to pool together scientific evidence for the ethnomedicinal uses of *D. tripetala*. A critique of the literature is provided, as well as suggestions for future studies that can pave the way for further discoveries on the medicinal effects of *D. tripetala*.

## 1. Introduction

**Figure 1:**
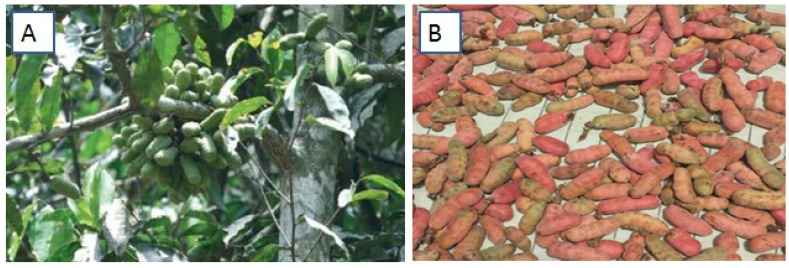
(**A**) *Dennettia tripetala* tree with leaves and unripe fruits. Image courtesy: World Agroforestry Center and Rubber Research Institute of Nigeria (**B**) Ripe (red) and unripe (green) *Dennettia tripetala* fruits.

*Dennettia tripetala*, hereafter referred to as DT, is also known as pepperfruit. It is widely grown in the rain forest zones of Nigeria and some parts of West Africa. DT is commonly consumed for its spicy taste. It is known in Nigeria by the following names: Ako (Edo), Mmimi (Ibo), Ata Igbere (Yoruba). It is also used in traditional medicine as a remedy for cough, fever, toothache, diarrhea, diabetes, and nausea in pregnant women. It grows as a small woody shrub. The tree can grow to a height of 12–15 m and have a girth of 0.6 m. The wood is white in color (Figure 1A) and soft [1,2,3]. The bark of DT possesses a very strong characteristic scent. The fruits are green when developing but start to turn red with ripening (Figure 1B). The moisture content also increases with ripening. The fruits possess a very strong characteristic smell. The leaves are 3–6 inches long and 1.5–2.5 inches broad. They are elliptic in shape. The fruits are mainly made up of the seeds and a bit of hard, spicy flesh. The fruit and seeds are edible and are consumed because of the spicy nature. The wood is used as fuel. The plant usually produces fruit between the months of March and May. For this reason, local traders preserve the seeds of pepperfruit by drying it under the sun in order to ensure continuous availability until the next harvest [1,2,3].

## 2. Classification of *Dennettia tripetala*

*Dennettia tripetala* is classified as follows:

Kingdom: Plantae; Phylum: Magnoliophyta; Class: Magnolidae; Order: Magnoliales; Family: Annonaceae; Genus: Denettia; Species: *Dennettia tripetala*.

## 3. Research on *Dennettia tripetala*

DT has received minimal scientific attention and this review aims to bring together a summary of the major work done so far while providing critique and suggestions for future work.

### 3.1. Biochemical Composition and Nutritive Value of Dennettia tripetala

The gas chromatography-mass spectrometry technique has been employed in the analysis of the essential oil of DT seeds obtained using the simultaneous distillation extraction technique. Twenty-five compounds were identified in the *n*-hexane seed extract, including linoleic acid ethyl ester, caryophyllene, 3-carene, phenyl ethyl alcohol, and cubebene. Phytochemical screening of the ethanolic extract revealed the presence of tannins, alkaloids, steroids, flavonoids, cardiac glycosides, saponins, and terpenoids [4]. These constituents provide a scientific basis for the use of DT in traditional medicine. Saponins, tannins, and flavonoids, for instance, are effective against diabetes. They also possess antimicrobial and anti-inflammatory properties [5,6]. Cardiac glycosides can be used in the treatment of asthma [7]. Alpha-linoleic acid has been shown to reduce the risk of cardiovascular disease [8] as well as prostate cancer in men [9]. Since these compounds are present in DT, there is the possibility of DT being useful in the treatment of these diseases; unfortunately, there is a dearth of literature in this area. Few preliminary studies have attempted to determine the nutritive value of DT. Researchers have shown that the fruit of DT when dry majorly consists of carbohydrates [3,10]. DT also contains protein, fiber, ash, lipids, and moisture. In fresh fruits, this moisture increases with ripening [10]. DT also contains trace elements, minerals, and water-soluble vitamins [3,10]. These vitamins A and C tendto increase significantly with ripening [10]. Ihemeje and colleagues [10] also discovered that, with ripening, the amount of phytochemicals, including phenols, saponins, tannins, flavonoids, and alkaloids, in DT changes.

### 3.2. Antimicrobial Properties of Dennettia tripetala

Researchers at Delta state University, Abraka, have found that the essential oil and phenolic acid extract of DT can inhibit the growth of food-borne microorganisms such as *Staphyloccocus aureus*, *Salmonella* sp., *Escherichia coli*, and a host of others [1]. This points to a role for pepperfruit in the preservation of food substances such as meat which is prone to rapid decomposition in places without constant electricity. More recently, the leaves of DT were found to be effective in inhibiting the growth of the rot-causing fungus *Sclerotium rolfsii* in cocoyam both *in vitro* and *in vivo* [11]. Several other reports show the antimicrobial activity of DT [12,13].

### 3.3. Analgesic and Anti-Inflammatory Effects of Dennettia tripetala

The essential oil of DT fruits has been found to possess analgesic effects as great as that induced by the powerful opioid morphine as well as aspirin and indomethacin. This oil also relieved inflammation in rodents with edema to levels comparable with that of dexamethasone [14]. The mechanism by which DT exhibits its analgesic effects was inferred by the fact that Naloxone, which inhibits the analgesic effect of morphine, was also able to inhibit that of DT. This result backs up the use of DT in pain and fever in folk medicine.

### 3.4. Other Effects of Dennettia tripetala on the Nervous System

More recently, researchers have discovered a component of the essential oil of the fruits, leaves, and seeds of DT which is largely responsible for the observed neuropharmacological effects of the oil. This compound, 1-nitro-2-phenyl ethane, exhibits hypnotic, anticonvulsant, and anxiolytic effects in mice [14].

### 3.5. Antihyperglycemic Effect of Dennettia tripetala

Recent research has provided evidence and a preliminary mechanism for the antihyperglycemic effect of the ethyl acetate extract of DT. Anaga and Asuzu in 2010 [15] showed that DT can reduce the plasma glucose level in drug-induced hyperglycemic rats to levels comparable with that of normal rats. This effect was found to be more pronounced than that of Tolbutamide. Using 3T3-L1 adipocytes and brefeldin, these same researchers investigated the possible mechanism for this observed phenomenonin DT. It was found that DT exerts this effect partly by recruiting glucose uptake proteins from the interior of the cell to the plasma membrane [16].

### 3.6. Antioxidant Effect of Dennettia tripetala

In living organisms, reactive oxygen species (ROS) are generated as a part of metabolism. These ROS are usually hindered from causing oxidative damage to cellular constituents by antioxidants present in the organism. Some of these antioxidants are produced in the body in the form of antioxidant enzymes, while others have to be consumed from plants in the form of antioxidant nutrients [17].

Preliminary phytochemical analysis has revealed the presence of antioxidants such as flavonoids and ascorbic acid in DT [10]. Recently, a group of researchers at the Federal University of Technology, Akure, evaluated the changes in antioxidant content and potentials of fresh DT fruits with ripening. Using the aqueous extract, they found that the phenol content increased with ripening, while the ascorbic acid and flavonoid content did not change [18]. Intriguingly, their results showed that the aqueous extract of unripe DT possesses greater antioxidant ability compared to ripe DT as typified by higher reducing power, greater ability to scavenge ABTS, DPPH, and OH, as well as higher Fe reducing and chelating potential [18]. They therefore concluded that, as DT undergoes ripening, its total phenol content increases, but its antioxidant potentials decreases. In 2011, a different group of researchers isolated two flavonoid glycosides from the ethyl acetate fraction of a 20% aqueous methanol extract of DT leaves which were found to instantly bleach the purple color of DPPH, indicating free radical scavenging potential [19]. In 2014, researchers at the University of Benin evaluated the antioxidant activity of the roots of DT. They discovered that the ethanolic extract of DT roots exhibits the ability to reduce ferric ion in a concentration-dependent manner [20]. The extract also possesses a H_2_O_2_-scavenging ability similar to that of ascorbic acid. Furthermore, the extract inhibits lipid peroxidation in frozen animal samples to an extent comparable with that of vitamins C and E [20]. They therefore concluded that DT may be useful in the preservation of frozen meat.

Other *in vitro* antioxidant studies have been done using the methanol extract of DT leaves and results show that DT possesses strong antioxidant potentials *in vitro* [21].

From the preceding paragraphs, it is obvious that a lot of investigations have been carried out on various parts of DT using a wide range of solvents, but to the best of my knowledge, there has been no attempt to confirm the antioxidative potentials of this plant *in vivo*.

### 3.7. Toxicity of Dennettia tripetala

A number of studies have been carried out to ascertain the toxicity of DT. Although DT has been reported to contain uvariopsin, an alkaloid which improves bile secretion and attenuates hepatic disorders [22,23], a study by Ofem and colleagues [22] showed that the ethanolic extract of DT fruits administered at a certain dose reduces bile production in normal healthy rats. The extract also caused an increase in sodium, potassium, and bicarbonate ions in bile and reduced the chloride and unconjugated bilirubin content of bile [22].

The effect of the ethanolic extract of DT on hematological parameters in normal healthy rats has also been investigated by Ikpi and Nku [24]. Firstly, they carried out an acute toxicity test to determine theLD_50_ of DT and a moderately high value of 251.19 g/kg·bw was gotten when the ethanolic extract of DT was administered intraperitoneally to normal healthy mice. Subsequently, they administered DT in normal saline orally to normal healthy rats and observed that, at low to moderate dose, DT may be hematotoxic to rats. Interestingly, the observed toxicity seemed to be relieved when the dose of DT administered was increased [24].

The toxic effects of the ethyl acetate root extract of DT have also been studied. An LD_50_ value of1120mg/kg was gotten from the intraperitoneal administration of the extract [25]. Although, the extract exhibited mild toxicity on the liver, kidney, spleen, and blood cells, it was seemingly beneficial to the hearts of mice following prolonged exposure [25].

The hexanolic extract of DT fruits has been found to be toxic to the larvae of the *Aedes aegypti* mosquito and this points to the potential for generating insecticides from *Dennettia* essential oil [12].

### 3.8. Effect of Dennettia tripetala on Healthy Humans

The seeds of DT have been found to be effective in reducing the intraocular pressure of normotensive emmetropic humans [26]. This suggests that DT could be put to use in the possible prevention and management of glaucoma.

### 3.9. Effect of Dennettia tripetala on Cancer

A recent report from the University of Illinois at Chicago showed that DT extract inhibits the growth of prostate cancer cells [27]. In the study, the ethanolic extract of DT seeds was tested for its efficacy on prostate cancer cell lines PC3 and LNCaP. The extract of DT was found to possess growth-inhibitory and cytotoxic effects on the prostate cancer cell lines *in vitro* [27].

## 4. Critique and Suggestions for Future Work

This review is aimed at bringing together the existing body of literature on *Dennettia tripetala*. A thorough search of the literature revealed that the body of scientific knowledge on *D. tripetala* is limited despite its use in African traditional medicine in alleviating a wide range of diseases.

There have been a few promising studies (as summarized above) that have yielded some interesting results on the phytochemical composition of the various extracts of the plant, although no follow-up work for many of these studies has been published. The authors tend to infer that the plant has certain medicinal potentials due to its content of specific phytochemicals. These studies can be improved by isolating specific components of the plant extract and investigating the effects of such components.

Such studies will provide a more logical basis for the conclusions that have been drawn prematurely. Another point to be critiqued is that, in most of the studies where the fruits of DT were used, the authors made no mention of the kind of fruits they used (ripe, unripe, or a mixture of both). It is important to specify this as the work by Ihemeje *et al.* [10] has shown that the different fruits possess a subtly different biochemical composition depending on the level of ripening. Another point that is noteworthy is that there are a good number of studies where the DT extract was administered orally to the animals in order to investigate the medicinal effects of DT, yet there is a lack of toxicity studies in which the extract was administered orally; the toxicity studies published so far were designed with intraperitoneal administration of the extract. This therefore introduces bias into any study where toxicity was investigated using a route of administration different from that used in investigating the medicinal potential of the extract.

From the literature review, there are very few *in vivo* studies on DT. Many of the conclusions drawn from *in vitro* studies need to be confirmed *in vivo*. The antioxidant properties of DT, for instance, are yet to be confirmed *in vivo*.

Another suggestion that is noteworthy is the fact that there is yet to be a study where DT (after being ground to a fine powder) is used to constitute a diet for animals to feed on directly. All the studies conducted so far have employed extracts using a wide range of solvents, but in reality, *Dennettia tripetala* fruits are consumed in West Africa in the natural raw form rather than in extracted forms. Thus, it will be interesting to see a study that is as close to reality as possible.

## 5. Conclusions

In conclusion, *Dennettia tripetala* has a lot of medicinal potential that is starting to be verified scientifically. Apart from the medicinal uses, this plant can also be put to use in the field of biotechnology as scientific evidence abounds for the potential use of this plant in meat preservation [1,20], pest control [11,12,28], and in food supplementation and spicing [3,10,29].
